# Artificial stem cells mediated inflammation-tropic delivery of antiviral drugs for pneumonia treatment

**DOI:** 10.1186/s12951-022-01547-x

**Published:** 2022-07-16

**Authors:** Aiping Qin, Sheng Chen, Songpei Li, Qizhen Li, Xiaotao Huang, Luoxing Xia, Yinshan Lin, Ao Shen, Andy Peng Xiang, Lingmin Zhang

**Affiliations:** 1 Key Laboratory of Molecular Target and Clinical Pharmacology and the State and NMPA Key Laboratory of Respiratory Disease, School of Pharmaceutical Sciences, The Third and The Fifth Affiliated Hospital, Guangzhou Medical University, Guangzhou, 511436 People’s Republic of China; 2 Department of Biomedical Engineering, Southern University of Science and Technology, Shenzhen, 518055 Guangdong China; 3 Center for Stem Cell Biology and Tissue Engineering, Key Laboratory for Stem Cells and Tissue Engineering, Ministry of Education, Sun Yat-Sen University, Guangzhou, 510080 China

**Keywords:** Artificial stem cells, Inflammation-tropic delivery, Pneumonia, Cytomegalovirus, Antiviral drugs

## Abstract

**Background:**

Cytomegalovirus (CMV) pneumonia is a major cause of morbidity and mortality in immunodeficiency individuals, including transplant recipients and Acquired Immune Deficiency Syndrome patients. Antiviral drugs ganciclovir (GCV) and phosphonoformate (PFA) are first-line agents for pneumonia caused by herpesvirus infection. However, the therapy suffers from various limitations such as low efficiency, drug resistance, toxicity, and lack of specificity.

**Methods:**

The antiviral drugs **G**CV and **P**FA were loaded into the pH-responsive nanoparticles fabricated by poly(lactic-co-glycolic acid) (**P**LGA) and 1,2-dioleoyl-3-trimethylammonium-propane (**D**OTAP), and further coated with cell **m**embranes derived from bone marrow mesenchymal stem cells to form artificial stem cells, namely **MPDGP**. We evaluated the viral suppression effects of MPDGP in vitro and in vivo.

**Results:**

MPDGP showed significant inflammation tropism and efficient suppression of viral replication and virus infection-associated inflammation in the CMV-induced pneumonia model. The synergistic effects of the combination of viral DNA elongation inhibitor GCV and viral DNA polymerase inhibitor PFA on suppressing the inflammation efficiently.

**Conclusion:**

The present study develops a novel therapeutic intervention using artificial stem cells to deliver antiviral drugs at inflammatory sites, which shows great potential for the targeted treatment of pneumonia. To our best knowledge, we are the first to fabricate this kind of artificial stem cell to deliver antiviral drugs for pneumonia treatment.

**Graphical Abstract:**

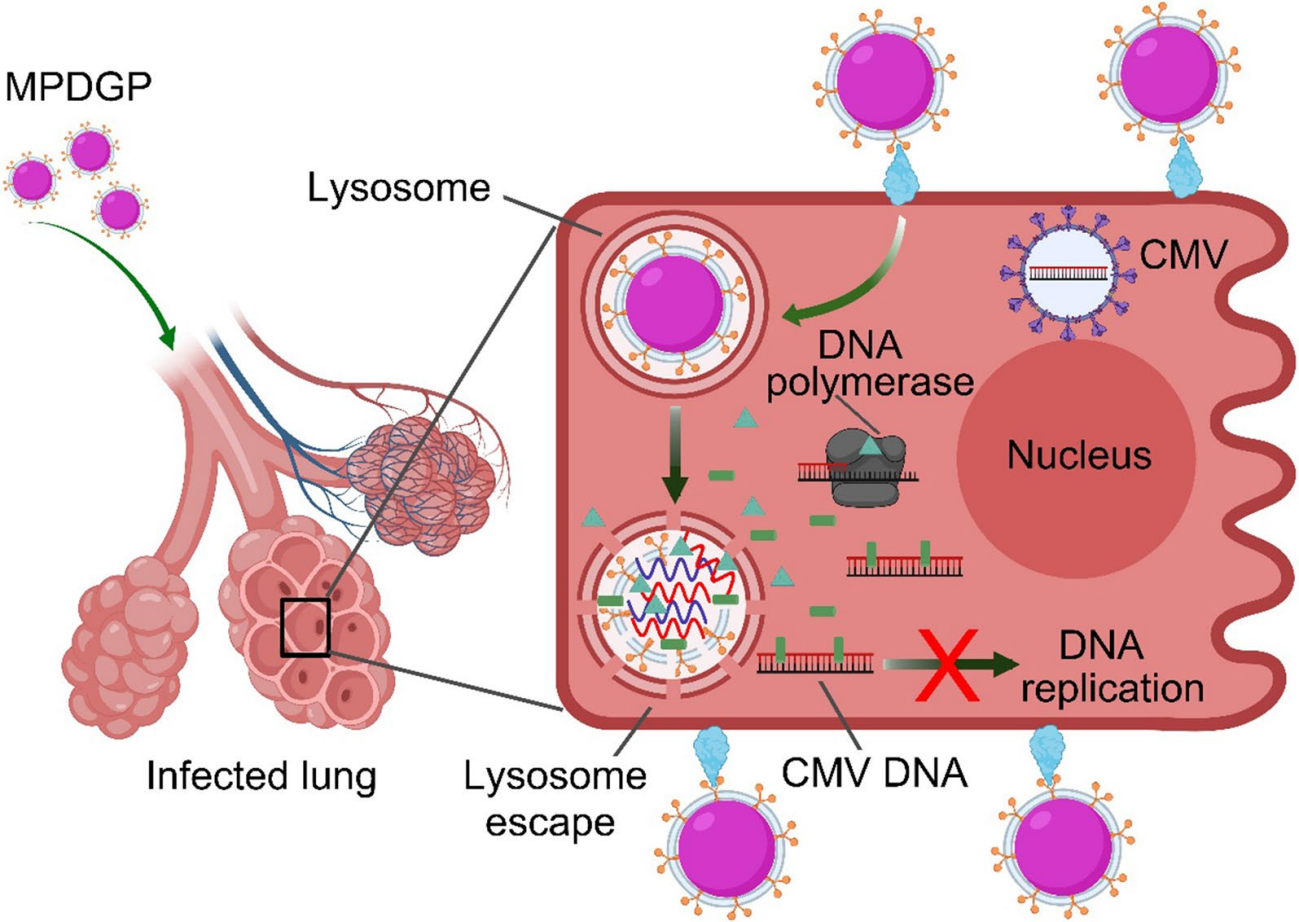

**Supplementary Information:**

The online version contains supplementary material available at 10.1186/s12951-022-01547-x.

## Background

Respiratory infections are a major public health problem and the leading cause of morbidity and mortality worldwide. Viral pathogens significantly contribute to pneumonia, a common and severe infection that can cause a tremendous global health burden, with COVID-19 pneumonia as the latest example [1, 2]. Cytomegaloviruses (CMVs) are species-specific viruses that belong to the β herpesvirus subfamily. CMV infection is often symptomatic and self-limited in healthy subjects. However, in immunocompromised individuals, such as transplant recipients or AIDS patients, CMV infection leads to life-threatening pneumonia, sight-threatening retinitis, and other serious problems [3, 4]. In addition, CMV infection is a common form of congenital infection that may induce intrauterine fetal death or hearing loss, brain damage, or neurodevelopmental delay [5, 6].

Current treatment strategies for CMV infection involve administering antiviral drugs and alleviating immunosuppression. Ganciclovir (GCV), the therapy of choice for CMV infections, and phosphonoformate (PFA), a drug to treat GCV-resistant CMV infections, are the two most widely used antiviral drugs against herpesvirus infection. Dosage recommendations for GCV for adult transplant patients with impaired renal function (Creatinine Clearance (mL/min) > 70) is less than 5.0 mg/kg q12 h [7], while 90 mg/kg q12 h for PFA [8]. However, the direct administration of these drugs may lead to acute organ injury and drug resistance, which counteracted their therapeutic effect [5, 9]. For example, a previous report indicated that the blood concentration of antiviral drugs reduced to ~ 15% from the peak concentration within 8 h post intravenously administration [10], which may decrease the antiviral effect significantly. Novel strategies for the treatment of severe CMV pneumonia are in great demand.

The advances in stem cell therapy offer an alternative strategy for treating pneumonia. The therapeutic effects of mesenchymal stem cells (MSCs) on pneumonia were mainly attributed to MSCs’ regenerative and immunomodulatory properties, which were confirmed by previous work [11]. The well-known pulmonary “first-pass” effect of MSCs after systemic administration may also favor treating lung disease using MSCs [12]. Moreover, MSCs possess inherent inflammatory migratory properties, mainly due to the various membrane receptors on their surfaces [13]. However, MSCs therapy also showed some limitations, such as the risk of pulmonary embolism induced by the micron-scale size, lack of large-scale supply of MSCs with “stem” properties, and when considering live cells as a delivery system, a limited drug loading capacity.

Cell membrane-camouflaged nanoparticles have emerged as promising drug delivery systems [14–16]. The cell membrane coating endowed the nanoparticles with cell-like functions, which increased the circulating time of nanoparticles or conferred immunocompatibility and targeting capacity to the nanoparticles [17, 18]. Inspired by cell membrane camouflage technology, we evaluated whether MSCs membrane-coated nanoparticles can serve as an efficient drug delivery system for treating pneumonia. To our best knowledge, these MSCs membrane-coated nanoplatforms for pneumonia treatments have not been reported yet.

**Scheme 1:**
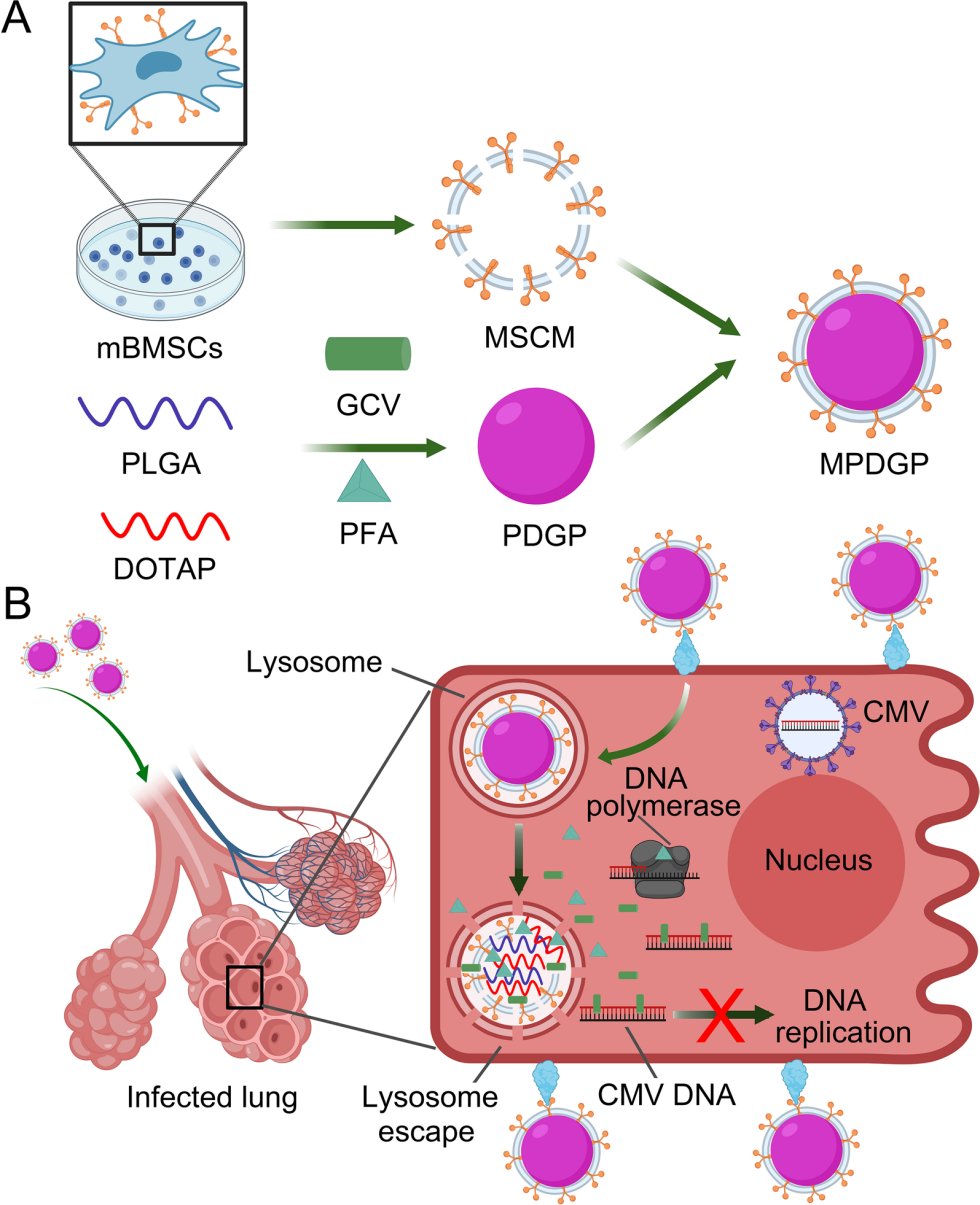
Scheme illustration of biomimetic nanoparticles for the therapy of pneumonia. **A** The preparation of MPDGP; **B** The treatment with MPDGP for CMV induced pneumonia. *mBMSCs* mouse bone marrow mesenchymal stem cells, *PLGA* poly(lactic-co-glycolic acid), *DOTAP* 1,2-dioleoyl-3-trimethylammonium-propane, *GCV* ganciclovir, *PFA* phosphonoformate, *PDGP* PLGA/DOTAP/GCV/PFA, *MPDGP* MSCM/PLGA/DOTAP/GCV/PFA, *CMV* Cytomegalovirus

Herein, we fabricated a novel style of artificial stem cells capable of loading the antiviral drugs, namely MPDGP. MPDGP was constructed with Food and Drug Administration-approved polymer poly(lactic-co-glycolic acid) (PLGA), combined with 1,2-dioleoyl-3-trimethylammonium-propane (DOTAP) to load the antiviral drugs (GCV and PFA), and further camouflaged with cell membranes derived from mouse bone marrow-derived MSCs (mBMSCs) (Scheme 1A). This style of biomimetic nanoparticles was expected to improve the targeted delivery and controlled release of drugs at inflammatory sites. Furthermore, we investigated the therapeutic effect of this type of artificial stem cell both in vitro and in vivo (Scheme 1B). The outcomes of testing this novel drug delivery system for CMV pneumonia treatments may also provide great potential for dealing with other lung infections, such as COVID-19 pneumonia.

## Materials and methods

### Virus and cell culture

Mouse Cytomegalovirus (MCMV) provides a valuable model for viral pathology since it is closely related to human CMV. MCMV (MCMV-GFP, Smith strain) was kindly provided by Dr. Minhua Luo from the Wuhan Institute of Virology, China. NIH 3T3 and MLE12 cell lines were cultured in DMEM and DMEM/F12, supplemented with 10% fetal bovine serum (FBS) (Gibco, USA), 1% Pen-Strep (100 U/ml penicillin, 100 μg/ml streptomycin) and 1% glutamine, respectively.

MCMV was grown and purified on NIH 3T3 cells. Briefly, NIH 3T3 cells were seeded in 15-cm dishes in complete DMEM and were infected with MCMV at a multiplicity of infection (MOI) of 0.1 for 3–4 days. The culture supernatants were then passed through a 0.45 μm filter, and virus particles were pelleted by ultracentrifugation through a 30% sucrose cushion (25,000 rpm for 3 h at 4 °C, Beckman SW32Ti rotor). Then, aliquots of the virus were kept at − 80 °C, and viral titers were measured by plaque assay in NIH 3T3 cells. Ten-fold serial dilutions of MCMV were added to NIH 3T3 cells of overnight culture for 3–6 h. Cell medium was removed and replaced with overlay medium supplemented with agarose, and the cells were further cultured at 37 °C for 4–5 days. And plaque-forming unit (PFU) was counted, which were identified by GFP expression using a Leica DMi8 microscope (Leica, Wetzlar, Germany).

### Isolation, culture, and identification of mBMSC

MSCs were isolated from the bone marrow of 4 ~ 6-week C57BL/6 mice under aseptic conditions. Briefly, bone marrow cells were separated and collected by flushing the bone marrow cavity from femurs and tibiae of mice. After red blood cells were lysed, the remaining cells were resuspended and cultured in L-DMEM complete medium containing 10% FBS at 37 °C and 5% CO_2_ incubator for 8–10 days. MSCs of passage 5 were detected by FACS for MSC surface antigens CD29 (eBioscience, 17-0291-80), CD44 (eBioscience, 12-0441-81), CD105 (eBioscience, 12-1051-81), SCA-1 (eBioscience, 45-5981-82), hematopoietic stem cells and endothelial cell markers CD45 (BD Pharmingen, 550994), CD31 (BD Pharmingen, 553372), CD34 (BD Pharmingen, 560230) and their isotypes. These cells were confirmed as MSCs by validating that they could differentiate into osteoblasts and adipocytes. All of the mBMSCs used in this study were collected in passages 3 to 8.

### Preparation of antiviral-drug-loaded nanoparticles (PDGP)

PLGA/DOTAP nanoparticles loaded with antiviral drugs were synthesized by a double emulsification process, as previously reported [18]. In short, 2 mg of GCV (Macklin, China) and 2 mg of PFA (Macklin, China) were dissolved in 500 μL of sterile normal saline at the weight ratio of 1:1, and the solution was added dropwise to 1 mL of the methylene chloride solution containing 20 mg of PLGA (Sigma-Aldrich, USA) and different mass of DOTAP (Alabama, USA) (1, 3, 6, 9, 12, and 15 mg) during a probe sonicator (60 W, 2 s on,1 s off). Through the second sonication of 60 s, the mixture was quickly added to 2 mL of 2% PVA solution. The obtained product was added to 10 mL of 2% PVA solution and stirred for 5 h at room temperature to evaporate the organic solvent, and then centrifuged at 12,000 rpm for 30 min. The pellet was washed thrice and suspended in sterile normal saline.

### Fabrication of MPDGP

The mBMSCs were used to extract the cell membranes. In short, the mBMSCs were treated with 0.25% Trypsin–EDTA Solution (Life Technologies, USA) and collected. The cell membranes were extracted by the Membrane and Cytosol Protein Extraction Kit (Beyotime, China) according to the Manufacturer’s introduction. Specifically, the cells were scraped from the culture dish and centrifuged at 2000 rpm for 5 min to precipitate the cells, and then the supernatant was removed. The membrane protein extraction reagent A was added to resuspend the cells, and the cells were repeatedly freeze-thawed three times at liquid nitrogen and room temperature, followed by the centrifugation at 4 °C for 10 min at 700*g* to precipitate the nuclei and unbroken cells, the supernatant was collected into a new centrifuge tube, and the supernatant was centrifuged at 4 °C for 30 min at 14,000*g* to collect cell membranes.

PDGP was mixed with the mBMSCs membrane at a weight ratio of 1:1, according to our previous work [17, 18]. The mixture was sonicated for 5 min and extruded back and forth 15 times with a LiposoFast-Basic extruder (Avestin Inc., Ottawa, Canada) to obtain MPDGP.

### mBMSC membrane protein validation

Sodium dodecyl sulfate–polyacrylamide gel electrophoresis (SDS-PAGE) was used to characterize membrane proteins. In short, MPDGP was subjected to radioimmunoprecipitation analysis (RIPA) (Beyotime, China) with control of total mBMSC protein, mBMSC membrane, and MPDGP. The protein concentration was measured using the Pierce BCA protein assay kit (Life Technologies). All samples diluted with SDS-PAGE loading buffer (Invitrogen, USA) were boiled at 100 ℃ for 5 min. Then, samples of the same protein mass (30 μg/well) were resolved onto a 10% SDS-PAGE gel (Beyotime, China) by electrophoresis. The resulting gel was stained in Coomassie blue, washed, and then imaged with the Amersham Imager 600 system (GE Healthcare Life Sciences, USA).

### Size, zeta potential, and morphology

Prepare PDGP and MPDGP suspension in ultrapure water, respectively. The average particle size and polydispersity index (PDI) of PDGP and MPDGP were measured by Zetasizer (Nano ZS, Malvern). PDGP and MPDGP were stained with 1% uranyl acetate (JEM-2000FX, Hitachi) and then observed under a transmission electron microscope (TEM).

### In vitro release studies

MPDGP filled with DiD (Solarbio, China) and FITC (Beyotime, China) was used for this purpose. An aliquot of 2 mL of MPDGP was loaded into a dialysate tube (Molecular weight cutoff, 3500 Da) and immersed in 20 mL of dialysate with a given pH (pH 7.4 or 5.0). After incubation at room temperature for different time points (1, 2, 4, 6, 8, 10, 12, 24, 48, and 72 h), an aliquot of 10 mL dialysate was taken and added with 10 mL fresh dialysate. The ultraviolet intensity of the collected dialysate, which indicates the release of FITC and DiD from MPDGP was measured with an ultraviolet spectrophotometer.

### Cellular uptake

To study the cellular uptake, the fluorescence dyes, FITC and DiD were loaded into the PLGA/DOTAP core. MLE-12 cells were seeded into confocal culture dishes at a density of 1 × 10^5^ cells per well. When the cells grew to about 80% confluence, they were pretreated with TNF-α (Peprotech, USA, 10 ng/mL) and IFN-γ (Peprotech, USA, 10 ng/mL) for 24 h. The cells were washed and incubated in a freshly prepared Opti-mem medium containing MPD/DiD/FITC. The dosage of DiD or FITC was equivalent to 0.1% of the biomimetic nanoparticles, respectively. After a specified incubation time, the medium was discarded, and the cells were gently washed three times with PBS. The cell nuclei were stained with Hoechst and then washed with PBS repeatedly. Cell imaging was performed under a confocal laser scanning microscope (LSM 880, Zeiss). For FACS analysis, the adherent cells were separated from the culture plate with 0.25% trypsin–EDTA solution (Life Technologies, USA) and suspended in 50 μL PBS. The cell suspension was analyzed by FACS (ImageStreamX Imaging, Amnis Corporation, USA). In addition, cell uptake of MSCNP (80 μg/mL) overtime (4, 8, 12, and 16 h) was also performed as above.

### Lysosomal escape

FITC and DiD are loaded into the MPD to assess lysosomal escape. MLE-12 cells were pretreated with TNF-α (10 ng/mL) and IFN-γ (10 ng/mL) for 24 h. The MLE-12 cells were incubated with MSCM/PLGA/DiD/FITC or MPD/DiD/FITC for 4 h and 12 h. Next, the cells were washed three times and stained with LysoBlue (KeyGEN BioTECH, China) for 60 min. The cells were washed with PBS and imaged under CLSM (LSM880, Zeiss, Germany).

### In vitro cytotoxicity study

The vitality/cytotoxicity kit (Beyotime, China) was used to test the in vitro cytotoxicity of the nanoparticles. Briefly, MLE-12 cells were seeded into confocal dishes for a density of 1 × 10^5^/well. To evaluate the cytotoxicity of MPDGP, when the cells grew to 80% confluence, the medium was replaced with a fresh medium containing MPDGP, which included an antiviral drug in indicated concentration. Cells treated with PBS or 75% EtOH were used as negative or positive controls, respectively. After incubation for 48 h, cells were stained with a Viability/ Cytotoxicity kit and imaged under CLSM (LSM880, Zeiss, Germany).

### In vitro inflammation targeting

MLE-12 cells in confocal culture dishes at about 80% confluence were washed and incubated in DEME/F12 medium containing 10 ng/mL TNF-α and INF-γ for 24 h to manufacture an in vitro inflammation model. Then, after incubating with MPDGP and loaded with DiD and FITC, respectively, for a specified time. Samples were analyzed under CLSM and by FACS, as mentioned above.

### Viral inhibition in vitro

MLE-12 cells were cultured in complete DMEM/F12, which contained 10% fetal bovine serum, 100 units/mL penicillin, and 100 μg/mL streptomycin. Add the virus to MLE-12 cells (MOI = 0.1) to simulate cell infection, then add different concentrations of MPDGP and incubate for a certain time. Observe the fluorescence intensity of GFP carried by the virus and detect the expression of MIE1 (MCMV immediate-early gene 1) mRNA by quantitative real-time polymerase chain reaction (qPCR) to determine the inhibitory virus effect.

### RNA isolation, cDNA preparation, and qPCR

Total RNA was extracted using TRIzol reagent (Invitrogen), and complementary DNA (cDNA) synthesis was performed using PrimeScript RT Master Mix (Perfect Real Time) (Takara, Japan). The target gene was amplified by SYBR Green qPCR (Takara RR820L). Normalized analysis of mRNA expression in vivo and in vitro using the 2 –∆ΔCt method against GAPDH. The primers sequence used are:

MIE1

Sense: 5’-TGAGGTGACCCGCATCCCAGTG-3’.

Antisense: 5’-CGAGGAGCAGTGCCAGAAGAAGC-3’.

GAPDH

Sense: 5’- TGCACCACCAACTGCTTAGC-3’.

Antisense:5’- GGCATGGACTGTGGTCATGAG-3’.

### MCMV pneumonia model

All animals were purchased from Beijing Vital River Laboratory Animal Technology Co., Ltd. (Beijing, China). We raised the animals in a specific pathogen-free (SPF) environment. All in vivo studies were performed in accordance with the Institutional Authority for Laboratory Animal Care of Guangzhou Medical University. MCMV pneumonia model was created in 4 ~ 6-weeks-old BALB/c-nu female mice. Briefly, after general anesthesia, Mice were infected with 2 × 10^5^ PFU of MCMV in 40 μL saline through intratracheal administration. Then the mice were randomized grouped to receive one of the following treatments: (1) Saline group: MCMV pneumonia + tail vein injection of 200 μL saline to MCMV pneumonia mice; (2) MPDG group: MCMV pneumonia + tail vein injection of 200 μL MPDG; (3) MPDP group: MCMV pneumonia + tail vein injection of 200 μL MPDP; (4) GP group: MCMV Pneumonia + tail vein injection 200 μL GP; (5) PDGP group: MCMV pneumonia + tail vein injection 200 μL PDGP; (6) MPDGP Group: MCMV pneumonia + tail vein injection of 200 μL of MPDGP. Mice of all groups also received GCV or PFA (5 mg/kg), respectively.

### Viral inhibition in vivo and cytokine determination

After treatment, the lung tissue of each mouse was taken for determination of viral MIE1 mRNA via qPCR and expression of viral GFP protein via Western Blotting. The expression of cytokines TNF-α and IL-6 were also accessed via qPCR and ELISA (Neobioscience, China). The primers sequence used are:

MIE1

Sense: 5’-TGAGGTGACCCGCATCCCAGTG-3’.

Antisense: 5’-CGAGGAGCAGTGCCAGAAGAAGC-3’.

TNF-α

Sense: 5’-CCCTCACACTCAGATCATCTTCT-3’.

Antisense:5’-GCTACGACGTGGGCTACAG-3’.

IL-6

Sense: 5’-CCAAGAGGTGAGTGCTTCCC-3’.

Antisense:5’-CTGTTGTTCAGACTCTCTCCCT-3’.

GAPDH

Sense: 5’-CCGCGTTCTTCCATTTGTGT-3’,

Antisense: 5’-ACATGATTTCGCATTTCGTCAT-3’;

### Biodistribution in vivo

To detect the distribution in vivo, MPD/DiR was injected into the tail vein (DiR is equivalent to 0.1% weight of the MPD) and was tracked by the IVIS Lumina XRMS Series III (PerkinElmer, USA) at time points of 1, 6, 12, 24 and 48 h. The mice were sacrificed, and organs were imaged. The fluorescence was analyzed with Living Image V4.5.5 software.

### Histological analysis and immunofluorescence staining

Tissues were fixed in 10% buffered formalin and embedded in paraffin. The tissue sections were obtained from lung tissue and stained with hematoxylin–eosin (H&E). For immunofluorescence staining, the sections were rehydrated and washed in PBS, pretreated for 1 h at room temperature with protein block solution (Dako, Carpinteria, CA). After incubation with CD68 (Servicebio, GB14043, 1:200) and LY-6G (Servicebio, GB11229, 1:200) overnight at 4 °C, sections were washed and incubated with fluorescence-labeled secondary antibodies. After the nucleus was stained with Hoechst (Invitrogen, USA), samples were examined under the microscope.

## Results and discussion

### Synthesis and characterization of MPDGP

To fabricate the biomimetic nanoparticles, we used poly (lactic-co-glycolic acid) (PLGA), an FDA-approved drug delivery, combined with 1,2-dioleoyl-3-trimethylammonium-propane (DOTAP) (PLGA/DOTAP, PD) to load antiviral drugs (GCV and PFA) (PDGP) based on our previous study [18]. The incorporation of DOTAP provided the nanoparticles with pH sensitivity and controlled release within the cells [18]. To optimize the ratio of PLGA to DOTAP, we performed a cell viability analysis with cell counting kit-8 (CCK-8). We found that the ratio of PLGA to DOTAP at 10 to 3 showed low toxicity, even with a concentration of PD at 240 μg/mL (Additional file 1: Fig. S1). The loading efficiency of PD for both drugs was ~ 80% (Additional file 1: Fig. S2), and the loading capacity was ~ 10% (Additional file 1: Fig. S3). Our previous work indicated that the weight ratio of the cell membrane to the PLGA-based core at 1:1 showed an ideal structure [18, 19], which was adopted in the following experiments.

**Fig. 1:**
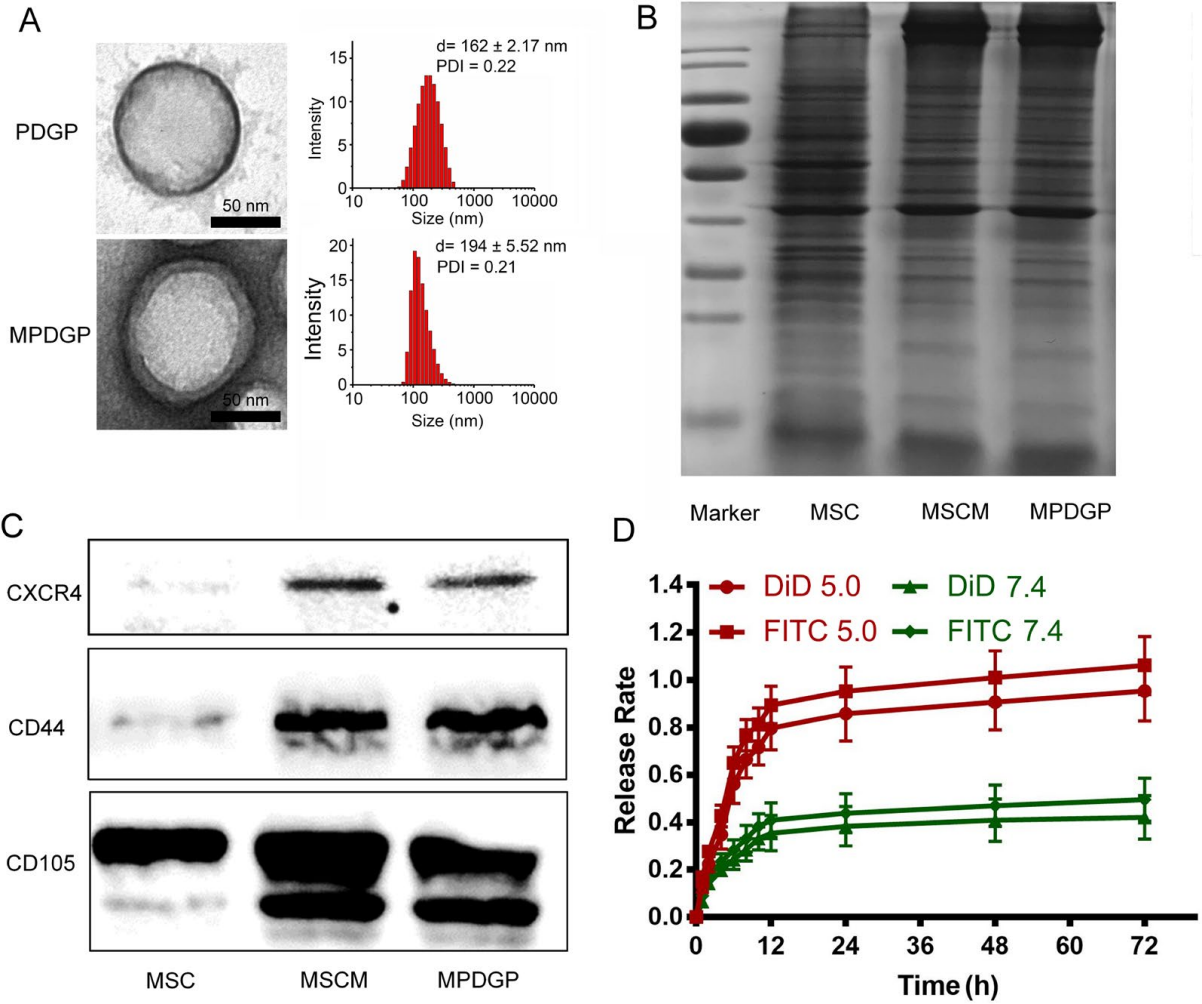
The characterization of bioinspired nanoparticles. **A** TEM and DLS analysis of nanoparticles. **B** SDS-PAGE analysis of protein compositions. **C** Western blotting analysis of cell markers. **D** The cumulative release of encapsulated fluorescence dyes (n = 3). PDGP, PLGA/DOTAP/GCV/PFA; MPDGP, MSCM/PLGA/DOTAP/GCV/PFA; MSCM, MSC membrane

Transmission electron microscope (TEM) analysis indicated that PDGP showed a spherical morphology, of which the size was ~ 162 nm as tested by dynamic laser scattering (DLS) (Fig. 1A). The cell membrane was extracted from mBMSC and identified by flow cytometry (FACS) analysis of mBMSC markers and endothelial cell markers, including CD29, CD44, SCA-1, CD105, CD31, CD34, and CD45 (Additional file 1: Fig. S4). The coating with mBMSC membrane formed a surrounding layer on the PDGP (MPDGP), as shown by TEM analysis (Fig. 1A and Additional file 1: Fig. S5), which increased the size of nanoparticles to ~ 194 nm examined with the DLS analysis, with the zeta potential decreasing from ~ 20 mV to ~ -20 mV (Additional file 1: Fig. S6). Noticeably, the hydrodynamic particle size is larger than the particle size under TEM because the samples for TEM analysis are in a dry state. In contrast, the ones for the DLS analysis in the hydrated state, as was confirmed by the previous work [20]. SDS-PAGE results indicated that MPDGP retained most of the membrane proteins of mBMSCs (Fig. 1B). Furthermore, the western blotting analysis showed the presence of antigens, including CXCR4, CD44, and CD45, on MPDGP nanoparticles (Fig. 1C). The previous report indicated that the proteins were nearly oriented in the right-side-out fashion entirely through the cell membrane camouflage technology, which ensured the cell membrane proteins functioning [21–23]. To evaluate how drugs were released from MPDGP, we used fluorescent dyes, fluorescein isothiocyanate (FITC) and 1,1'-dioctadecyl-3,3,3’,3’-tetramethylindodicarbocyanine perchlorate (DiD) to mimic GCV and PFA. The fluorescent profiles indicated that the release of both dyes from nanoparticles was higher in the acidic environment of pH 5.0 compared with the neutral one of pH 7.4 (Fig. 1D). At 72 h, the cumulative release ratio of DiD and FITC at pH 5.0 was over 80%, whereas, at pH 7.4, it was less than 50% (Fig. 1D). Although the use of sensitive fluorescence dyes would not completely show the release profile of drugs, the data confirmed the pH-responsiveness of the nanoparticles, which was useful in drug release after the cellular uptake. These data indicated that the biomimetic nanoparticles were fabricated and could release encapsulated components in an acidic environment. In addition, this mBMSC membrane camouflaged nanoplatform endowed the nanoparticles with a cell-like structure, which may inherit the circulating characteristics and tissue distribution properties.

### Cellular uptake

We studied the cellular uptake of the synthesized nanoparticles in the simulated inflammatory environment. Considering GCV and PFA can't be detected by the fluorescent method, we used FITC (green) and DiD (red) as indicators to track the cellular uptake of nanoparticles. We asked whether the cellular uptake was different between inflamed cells and uninflamed ones. With the increase of incubation time from 4 to 12 h, CLSM and FACS analysis indicated that the FITC/DiD dual positive cells increased significantly (Additional file 1: Fig. S7). In addition, the cellular uptake was approximately one-third higher in the inflammatory environment produced by TNF-α (10 ng/mL) and IFN-γ (10 ng/mL) compared with ones without cytokine stimulation (Additional file 1: Fig. S7). The evidence indicated that the biomimetic nanoparticles showed inflammatory tropism, which was consistent with the previous report, revealing that proinflammatory cytokine changed the structure and function of the cell membrane [24]. In the present study, we identified 624 differentially expressed proteins in inflamed MLE-12 cells, compared with untreated MLE-12 cells by proteomic analysis (Additional file 2: Appendix 1). In addition, TNFα has been shown to increase the permeability across the endothelial cell [25], contributing to the increased uptake of nanoparticles during inflammation. These differentially expressed proteins may be responsible for the selective uptake of nanoparticles, although we could not clarify which protein(s) may be the most critical. Our future study will investigate the detailed mechanisms underlying the uptake of nanoparticles in inflamed cells.

**Fig. 2:**
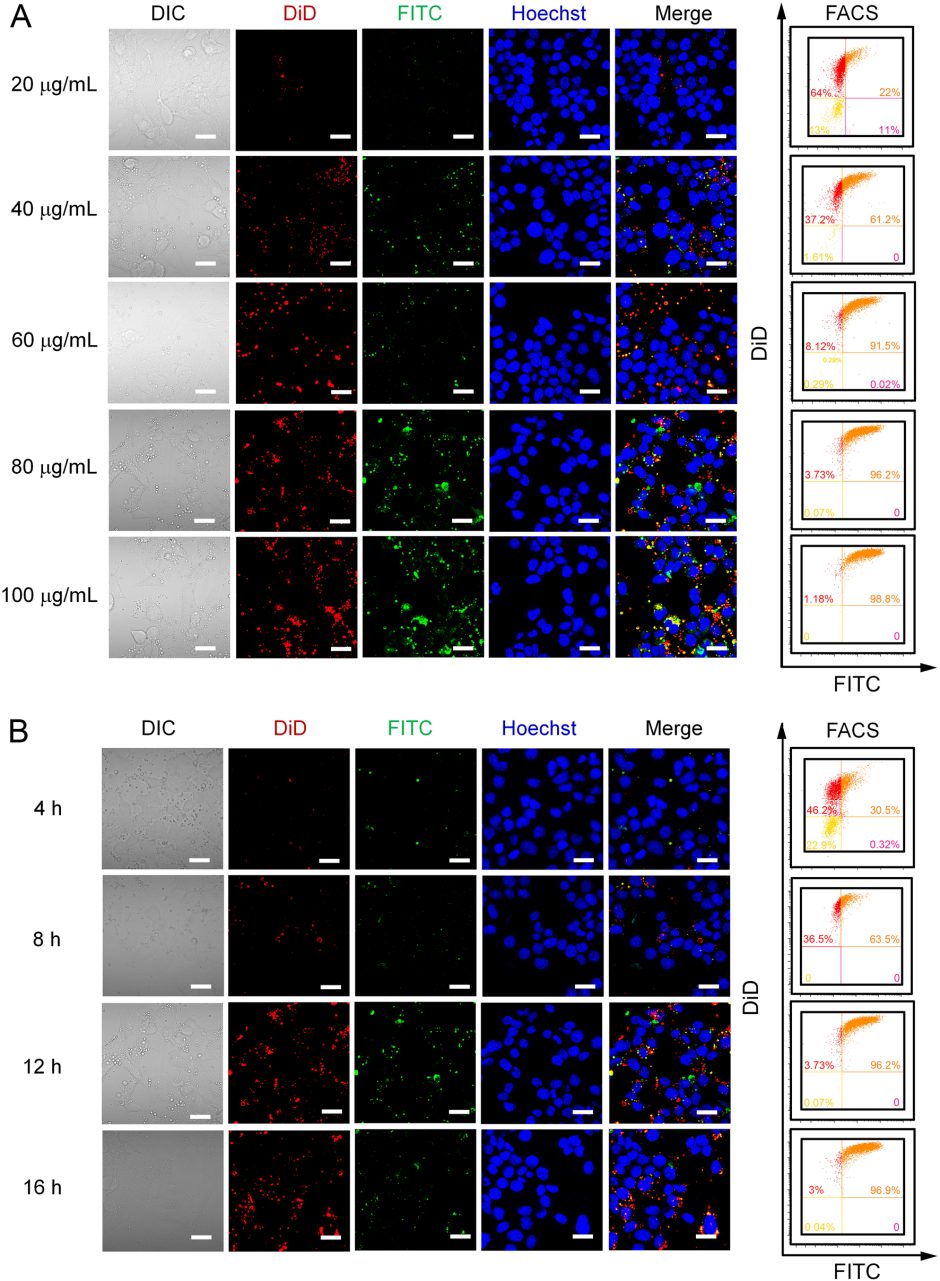
Cellular uptake of biomimetic nanoparticles. **A** Dosage-course of cellular uptake. MLE-12 cells were pretreated with TNF-α (10 ng/mL) and IFN-γ (10 ng/mL) for 24 h. MLE-12 cells were incubated with biomimetic nanoparticles in concentrations of 20, 40, 60, 80, and 100 μg/mL for 12 h. **B** Time-course cellular uptake. MLE-12 cells were incubated with biomimetic nanoparticles with a concentration of 80 μg/mL for 4, 8, 12, and 16 h, respectively. Scale bar, 20 μm

We further investigated the cellular uptake of a gradient concentration of biomimetic nanoparticles. Both CLSM and FACS analysis indicated that at a concentration of 80 μg/mL, the number of FITC and DiD dual positive cells reached a plateau, which was ~ 97% (Fig. 2A). We further analyzed the time course of cellular uptake. CLSM analysis indicated that most nanoparticles were taken up by the cells after being incubated for 12 h (Fig. 2B). Quantitative analysis by FACS showed that the FITC and DiD dual positive cells were more than 96% at 12 h. Further extension of incubation time did not increase the cellular uptake. Thus, this optimized condition, namely 80 μg/mL and 12 h, was adopted for further experiments. These data indicated that TNF-α and IFN-γ treatment facilitated the uptake of mBMSC camouflaged nanoparticles and the cellular uptake of biomimetic nanoparticles was dosage and time-dependent.

### The evaluation of anti-virus effect and cytotoxicity in vitro

**Fig. 3:**
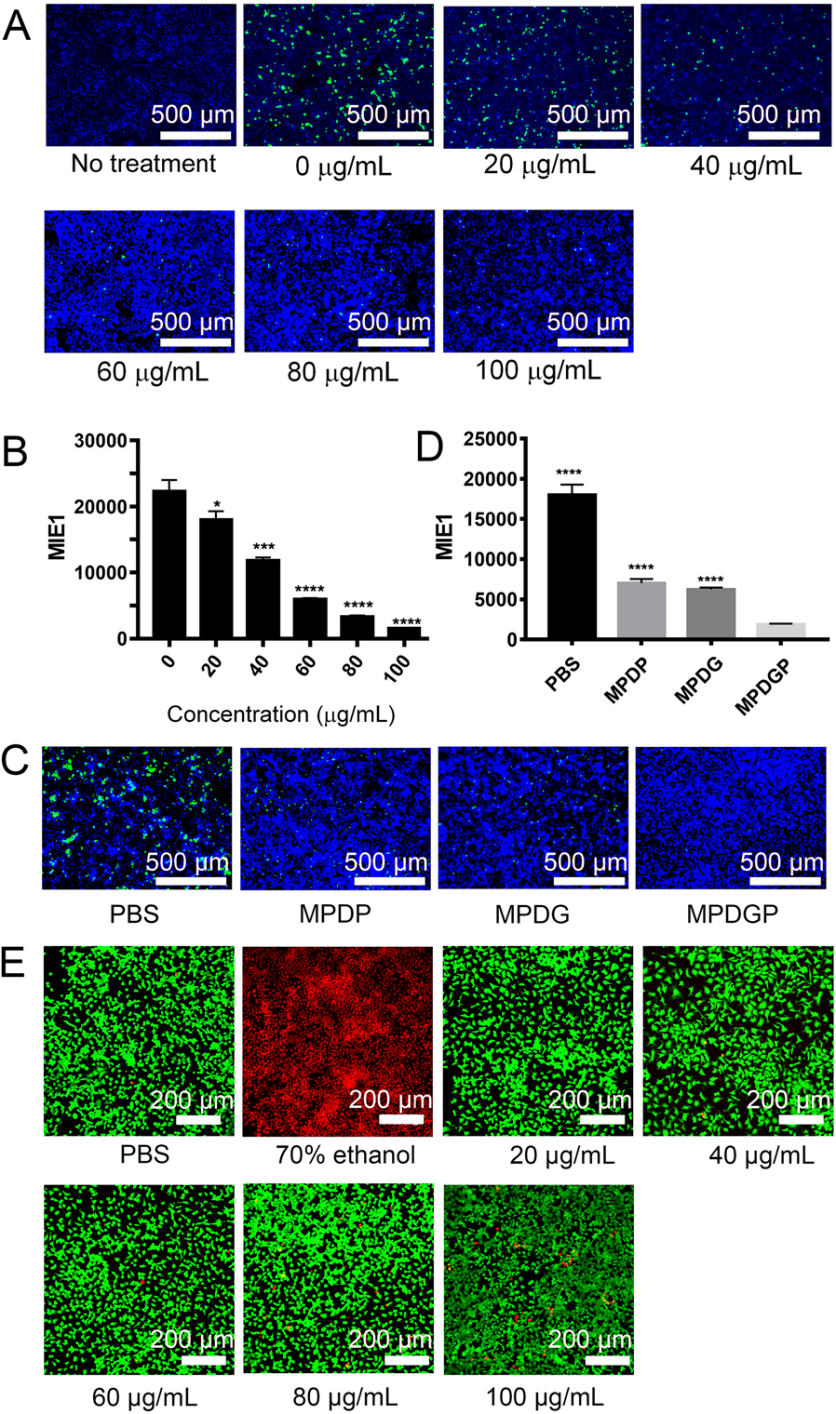
Exploration of dosing conditions. **A** Fluorescence image of viral GFP protein. **B** qPCR analysis of MIE1 level. Effect of different concentrations of drugs on the expression of MIE1 mRNA of the virus. **C** Fluorescence image of viral GFP protein. **D** qPCR analysis of MIE1 level. Effect of different drugs on the expression of MIE1 mRNA of the virus. **E** Live and dead staining image. For all graphs: *p < 0.05, ***p < 0.001, and ****p < 0.0001, n = 3, and values represent the mean ± SEM

We evaluate the antiviral and cytotoxic effect of these nanoparticles in CMV-GFP (Cytomegalovirus-green fluorescent protein) infected MLE-12 cells at a multiplicity of infection (MOI) of 0.1. Firstly, the effect on viral suppression was assessed by the measurement of GFP fluorescence. The nanoparticles at the dosage of 80 μg/mL showed a maximal antiviral effect, and a higher dosage of 100 μg/mL did not exert a more potent antiviral effect (Fig. 3A). Secondly, the expression of MCMV immediate early gene 1, namely MIE1 was detected by quantitative PCR (qPCR) analysis, which indicated that the dose-dependent inhibition of MIE1 level induced by biomimetic nanoparticles was consistent with the evaluation of GFP as a reporter (Fig. 3B). Thus, the dosage of 80 μg/mL was selected for further experiments. We also found that the treatment with MPDGP showed a more effective antiviral effect than the treatment with GCV or PFA along, and also MPD-encapsulated single-drug formulations (MPDG and MPDP) as indicated by the presence of GFP and the level of MLE1 (Fig. 3C, D). The live/dead assay was performed to assess the safety of the nanoparticles. CLSM analysis showed the nanoparticles no significant cytotoxicity at the working concentrations, which demonstrated the safety of the biomimetic nanoparticles (Fig. 3E). The above results indicated the practical antiviral effect of the synthesized nanoparticles with low cytotoxicity in vitro.

### Lysosome escape

**Fig. 4:**
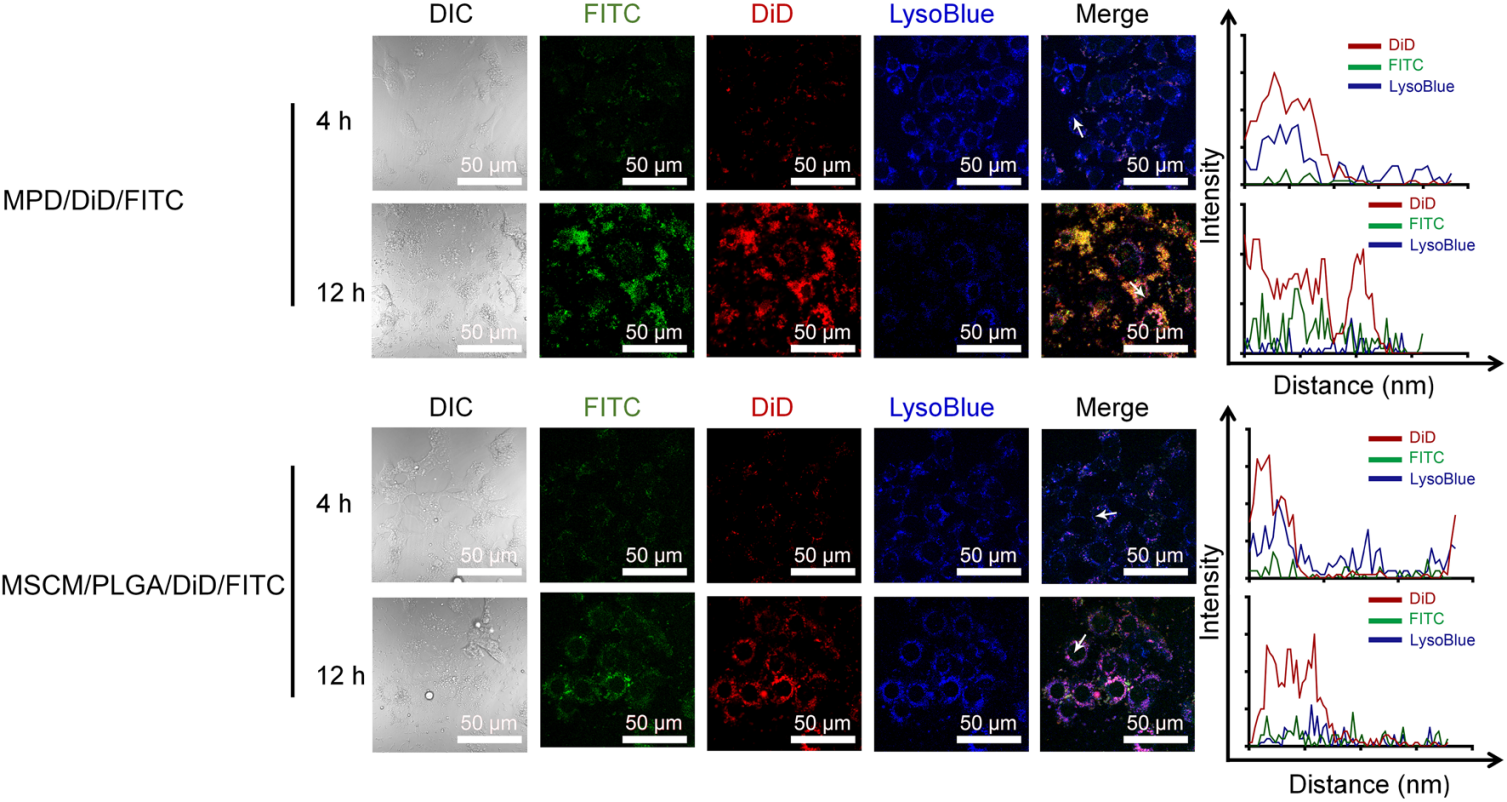
The evaluation of lysosome escape. MLE-12 cells were pretreated with TNF-α (10 ng/mL) and IFN-γ (10 ng/mL) for 24 h. After incubation with MPD/DiD/FITC or MSCM/PLGA/DiD/FITC for 12 h, the cells were stained with Lysotracker blue and analyzed with CLSM

Endocytosis is a primary mechanism of nanoparticles uptake, as indicated by previous reports [26, 27]. Lysosome escape is essential for the nanoparticle to exert desired cellular effects. In the present study, DOTAP-containing PLGA nanoparticles were synthesized, considering DOTAP has been shown to facilitate the lysosome escape of nanoparticles in previous reports from our and other's groups [18, 28]. We evaluated the lysosomal escape ability of delivery in vitro, which is an essential parameter in influencing the drug release [29–31]. The fluorescent dyes, FITC and DiD were used as model drugs, and the lysosomes were stained with lysotracker blue. After incubation for 12 h, the green and red fluorescence separated from the blue one in the MPD group (MPD/DiD/FITC) (Fig. 4, top). However, the MSCM/PLGA DiD/FITC without DOTAP showed an overlap of green, red, and blue fluorescence still (Fig. 4, down). We also quantified the lysosomal escape ability of the formulations by analyzing the co-localization between the components and lysosome, which indicated that the co-localization ratio of red/blue or green/blue induced by PLGA/DOTAP showed a significant decrease (from ~ 80% at 4 h to ~ 15% at 12 h), compared with the ones induced by PLGA (from ~ 80% at 4 h to ~ 50% at 12 h) (Additional file 1: Fig. S8). The blue fluorescence represented lysosomes reduced after the treatment with (MPD/DiD/FITC, which might be due to the addition of DOTAP improving the lysosomal escape by fusogenic property or proton sponge effect [18, 19]. To clear the concerns on the potential toxicity, we optimized the dosage of DOTAP, which showed cell viability over 80% when the ratio of PLGA to DOTAP was 10/3 (Additional file 1: Fig. S1) and adopted in the following experiments. Furthermore, the drug-loaded nanoparticles, MPDGP, also showed high cell viability without significant cell death examined by Live/Dead staining (Fig. 3E). The differences between MSCM/PLGA and MPD-based nanoparticles treated cells indicated that DOTAP facilitates the escape of nanoparticles from lysosomes.

### Biodistribution of biomimetic nanoparticles

**Fig. 5:**
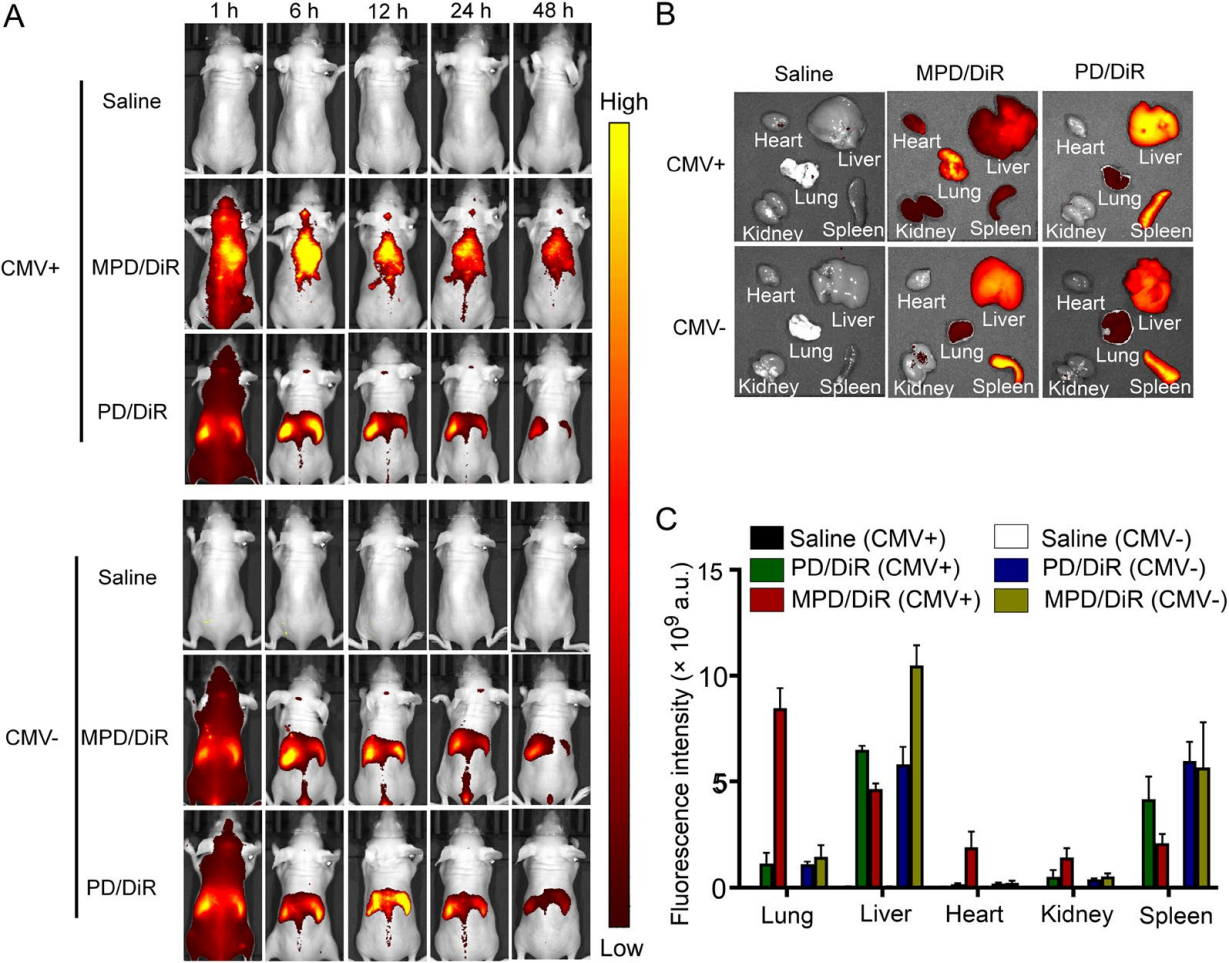
Exploration of in vivo targeting ability. **A** In vivo imaging images of each group of mice at different times. **B** In vivo imaging images of various organs. **C** The quantitative analysis of fluorescence intensity in various organs (n = 5)

Prerequisites for systemically administered nanoparticles to exert cellular activities include: (1) nanoparticles can reach target tissue; (2) nanoparticles can be taken up by the cells; (3) nanoparticles can escape from lysosomes. To demonstrate the biodistribution of the biomimetic nanoparticles, we used an in the vivo tracking system to monitor the DiR (1,1-dioctadecyl-3,3,3,3-tetramethylindotricarbocyanine iodide) labeled nanoparticles. The fluorescent images showed that the biomimetic nanoparticles MPD/DiR accumulated and retained in the lung of MCMV-infected mice during the observation period up to 48 h, indicating a specific delivery of antiviral drugs to the pneumonia sites (Fig. 5A). Conversely, no matter whether infected or not, the bare nanoparticles PD/DiR (without the camouflage of the mBMSC membrane) mainly accumulated in the liver, and the fluorescence signal quickly declined after 24 h (Fig. 5A).

The major organs, including the heart, liver, spleen, lungs, and kidneys, were examined individually. Ex vivo fluorescence showed that MPD/DiR nanoparticles mainly accumulated in CMV-infected lung tissues, with much less distribution in other organs, while most PD/DiR accumulated in the liver and spleen from CMV-infected mice (Fig. 5A, B). Consistent with in vivo observation, both MPD/DiR and PD/DiR accumulated in the liver and spleen of uninfected mice, with minimal pulmonary targeting. The quantitative analysis further confirmed the above results (Fig. 5C). These results indicated that the BMSC membrane camouflaged nanoparticles possessed inflammatory tropism properties in vivo.

### The effects of nanoparticles on viral suppression in vivo

**Fig. 6:**
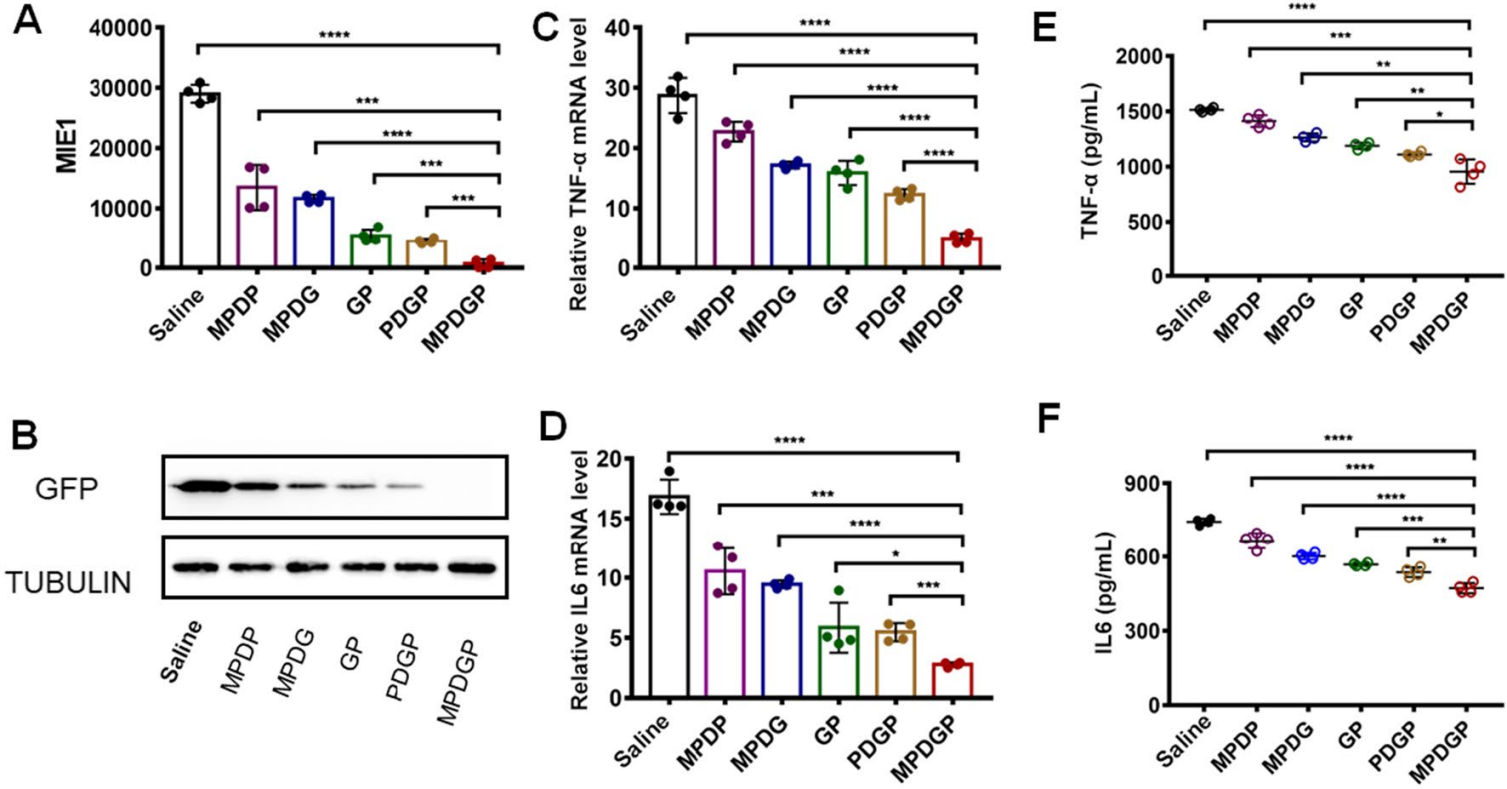
Virus suppression effect and changes in cytokine levels. **A** Viral MIE1 gene expression. **B** Viral GFP protein expression; **C**, **D** Lung TNF-α and IL-6 gene expression. **E**, **F** Lung TNF-α and IL-6 protein levels. (n = 4; *P < 0.05, **P < 0.01, ***P < 0.001, ****P < 0.0001). MPDP, MSCM/PLGA/DOTAP/PFA; MPDG, MSCM/PLGA/DOTAP/GCV; GP, GCV/PFA; PDGP, PLGA/DOTAP/GCV/PFA; MPDGP, MSCM/PLGA/DOTAP/GCV/PFA

We evaluated the effects of synthesized nanoparticles on viral suppression and proinflammatory cytokine levels. MCMV-infected pneumonia mice were treated with saline, MPD-encapsulated GCV or PFA alone (MPDG or MPDP), bare GCV and PFA (GP), naked nanoparticles loaded with GCV and PFA (PDGP), and MPD loaded with GCV and PFA (MPDGP). qPCR results showed the most significant inhibition of MIE1 in the lung tissue of mice treated with MPDGP compared with other groups, and the MIE1 level was approximately 100-fold lower than the saline-treated mice (Fig. 6A). Western blotting results also showed the elimination of the GFP signal in lung tissue completely, suggesting potent viral suppression induced by MPDGD (Fig. 6B). The anti-inflammatory effect of nanoparticles was evaluated by the expression level of proinflammatory cytokines, including TNF-α and IL-6. MPDGD treatment most effectively suppressed proinflammatory cytokines, led to a 90% or 86% reduction of TNF-α and IL-6 mRNA expression, respectively, when compared with the saline group (Fig. 6C and D). ELISA results further confirmed the reduction of the TNF-α and IL-6 from lung homogenate after MPDGD treatment compared with other groups (Fig. 6E and F).

**Fig. 7:**
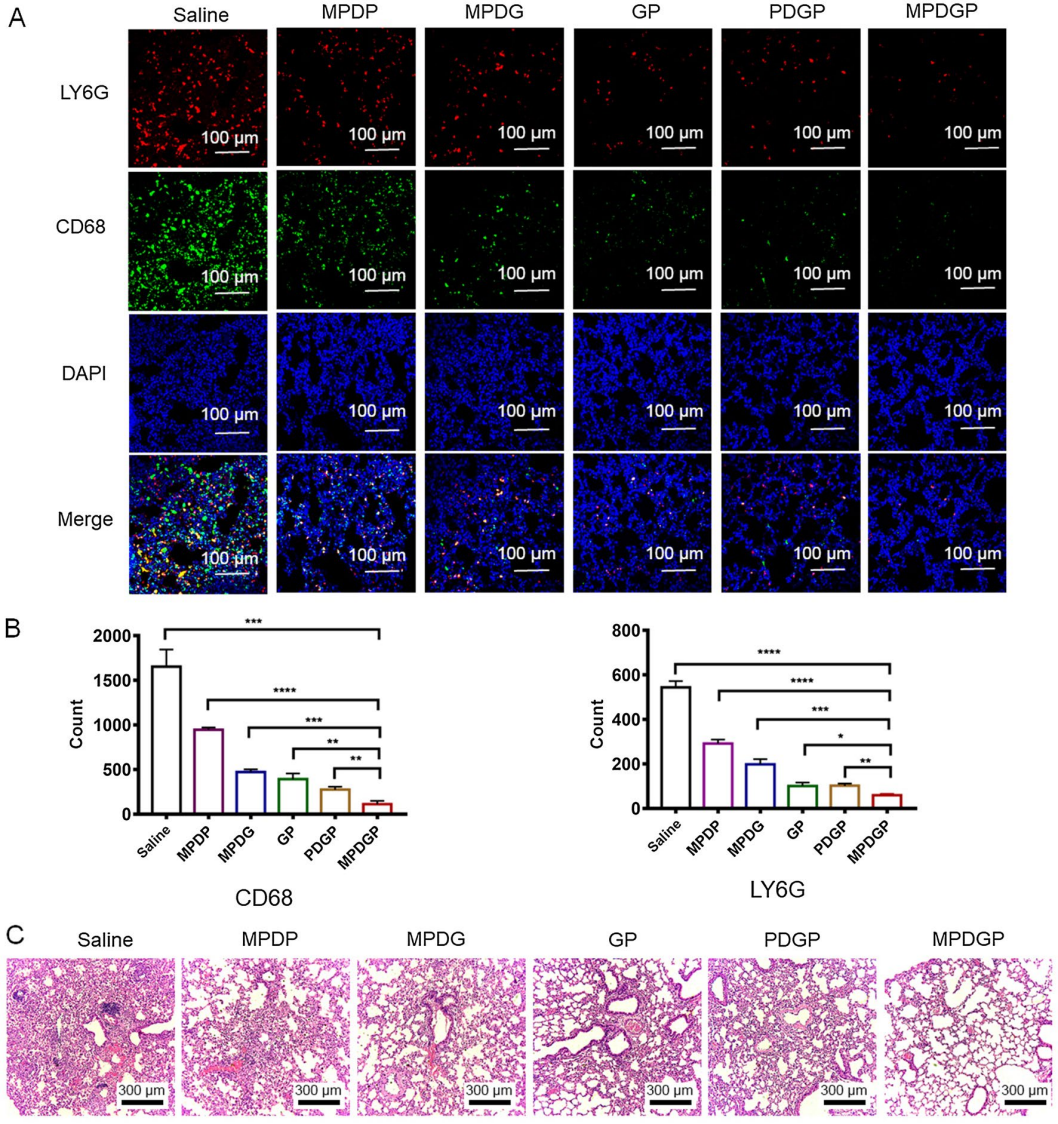
The effect of biomimetic nanoparticles on lung inflammation and histological severity of MCMV pneumonia. **A** CLSM analyzes of lung leukocyte infiltration. **B** Statistics of fluorescence number of each group (n = 5). **C** HE staining image of the lung. (n = 5, *P < 0.05, **P < 0.01, ***P < 0.001, ****P < 0.0001). MPDP, MSCM/PLGA/DOTAP/PFA; MPDG, MSCM/PLGA/DOTAP/GCV; GP, GCV/PFA; PDGP, PLGA/DOTAP/GCV/PFA; MPDGP, MSCM/PLGA/DOTAP/GCV/PFA

To investigate the effects of nanoparticles on inflammatory cell infiltration in the lungs, LY6G (a biomarker for granulocytes and neutrophils) and CD68 (a biomarker for macrophages) were detected by immunofluorescence. The results showed that MPDGP significantly reduced the number of LY6G or CD68 positive cells compared with other groups, indicating that MPDGP markedly suppressed the infiltration of inflammatory cells in the lung (Fig. 7A and B). These results revealed that the fabricated antiviral drug-loaded biomimetic nanoparticles inhibited MCMV infection-associated inflammation mainly by inhibiting inflammatory cell infiltration. In addition, histological analysis of the lung tissue showed MPDGP treatment significantly alleviated pulmonary histopathological changes, such as increased alveolar airspace and decreased alveolar tissue, compared with other groups (Fig. 7C). Although the strategy by MSCM mediated inflammation-tropic delivery showed effective inhibition of MCMV infection-associated inflammation, we considered that the MPDGP was also applicable in the immunocompromised or immunodeficient individuals suffering from CMV, which was evidenced by PDGP without inflammation-tropic property also showed considerable inhibition of MIE1 and the viral GFP expression (Fig. 6A and B). Taken together, the above results suggested that the MPDGP showed a better therapeutic effect on MCMV-infected mice compared with other groups.

## Conclusion

In conclusion, we have developed a novel style of artificial nanoparticles, which enable us to deliver antiviral drugs effectively to the inflamed lung. Based on the natural cell membrane camouflaged nanostructure, the nanoparticles tend to accumulate in the inflammatory tissues and facilitate the delivery of antiviral drugs for pneumonia treatment. Notably, we found that the synergetic inhibition of the viral DNA elongation and the activity of viral DNA polymerase suppressed the inflammation efficiently. Our work provides a novel strategy for CMV pneumonia treatments, which might be adapted easily for dealing with other lung infections, such as COVID-19 pneumonia.

## Supplementary Information

**Additional file 1: Fig. S1. **The screening of appropriate ratios of PLAG/DOTAP. The cells were treated with different nanoparticles for 48 h. **Fig. S2.** The loading efficiency of PLGA/DOTAP nanoparticles. The dosage of PLGA was 20 mg (n=3). **Fig. S3.** The loading capacity of PLGA/DOTAP nanoparticles. The dosage of PLGA was 20 mg (n=3). **Fig. S4**. The identifications of mBMSCs by FACS. (A) The morphology of mBMSCs at passages 5 (P5). (B) Adipocyte differentiation with Oil Red O staining. (C) Osteoblast differentiation with Alizarin red S staining. (D) FACS analysis of the surface markers of mBMSC. Among them, CD29, CD44, CD105, and SCA-1 were positively expressed, and CD45, CD31, and CD34 were negatively expressed. **Fig. S5**. TEM analysis of MPDGP. **Fig. S6** Zeta potential analysis of the nanoparticles. **Fig. S7.** Inflammatory tropism *in vitro*. MLE-12 cells were pretreated with or without TNF-α (10 ng/mL) and IFN-γ (10 ng/mL) for 24 h. The cells were treated with MDP/DiD/FITC for 4 h or 12 h. CLSM and FACS analysis were performed to evaluate the inflammatory tropism. **Fig. S8.** The co-localization ratios analysis between components and lysosomes.

**Additional file 2. **Proteomic analysis of the differential expression membrane proteins with or without inflammatory factor.

## Data Availability

All data generated or analyzed during this study are included in this published article and its supplementary information.

## Abbreviations

CLSM: Confocal laser scanning microscope
DLS: Dynamic light scattering
FITC: Fluorescein isothiocyanate
qPCR: Quantitative PCR
TEM: Transmission electron microscopy
TNF-a: Tumor necrosis factor-a
IL-6: Interleukin-6
PLGA: Poly(lactic-co-glycolic acid)
DOTAP: 1,2-Dioleoyl-3-trimethylammonium-propane
DiD: 1,1’-Dioctadecyl-3,3,3’,3’-tetramethylindodicarbocyanine perchlorate
MCMV: Murine cytomegalovirus
MSC: Mesenchymal stem cell
GCV: Ganciclovir
PFA: Phosphonoformate
GFP: Green fluorescent protein
PFU: Plaque forming unit
MOI: Multiplicity of infection
FBS: Fetal bovine serum
