# Supplementary material for: Artificial stem cells mediated inflammation-tropic delivery of antiviral drugs for pneumonia treatment
Source: J Nanobiotechnology. 2022 Jul 16;20:335. doi: 10.1186/s12951-022-01547-x (PMC9287715; doi:10.1186/s12951-022-01547-x)
Supplement: Supplementary file 1 — Additional file 1: Fig. S1. The screening of appropriate ratios of PLAG/DOTAP. The cells were treated with different nanoparticles for 48 h. Fig. S2. The loading efficiency of PLGA/DOTAP nanoparticles. The dosage of PLGA was 20 mg (n=3). Fig. S3. The loading capacity of PLGA/DOTAP nanoparticles. The dosage of PLGA was 20 mg (n=3). Fig. S4. The identifications of mBMSCs by FACS. (A) The morphology of mBMSCs at passages 5 (P5). (B) Adipocyte differentiation with Oil Red O staining. (C) Osteoblast differentiation with Alizarin red S staining. (D) FACS analysis of the surface markers of mBMSC. Among them, CD29, CD44, CD105, and SCA-1 were positively expressed, and CD45, CD31, and CD34 were negatively expressed. Fig. S5. TEM analysis of MPDGP. Fig. S6 Zeta potential analysis of the nanoparticles. Fig. S7. Inflammatory tropism in vitro. MLE-12 cells were pretreated with or without TNF-α (10 ng/mL) and IFN-γ (10 ng/mL) for 24 h. The cells were treated with MDP/DiD/FITC for 4 h or 12 h. CLSM and FACS analysis were performed to evaluate the inflammatory tropism. Fig. S8. The co-localization ratios analysis between components and lysosomes. [file 12951_2022_1547_MOESM1_ESM.docx]

**Additional information**


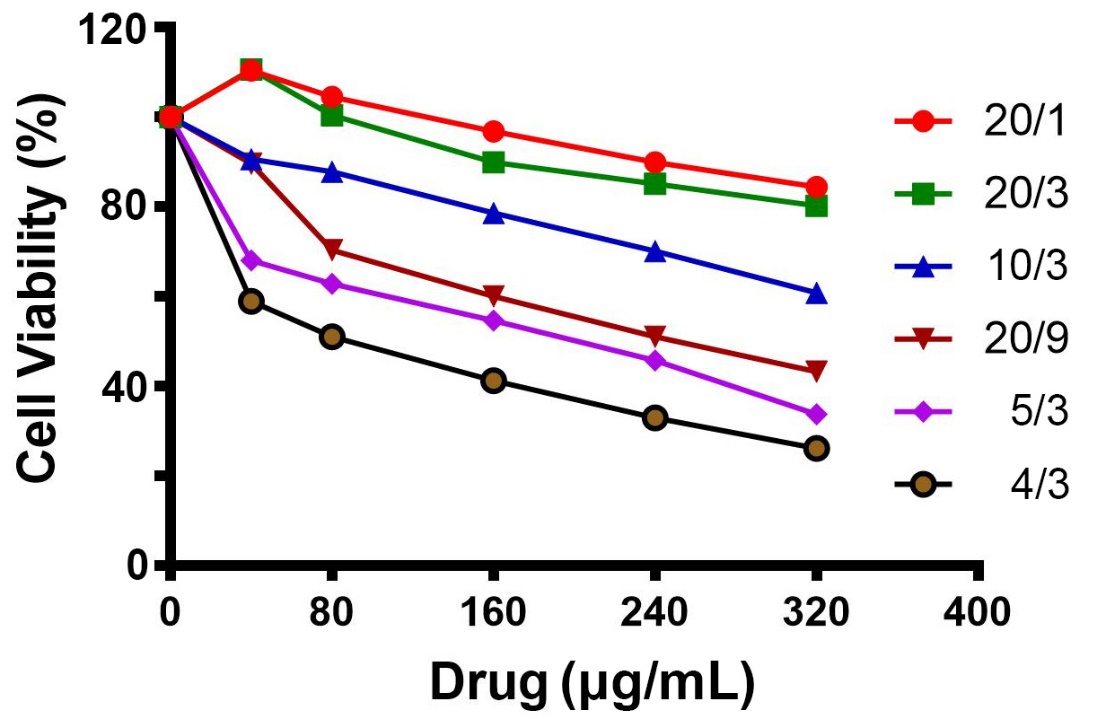


**Fig. S1.** The screening of appropriate ratios of PLAG/DOTAP. The cells were treated with different nanoparticles for 48 h.


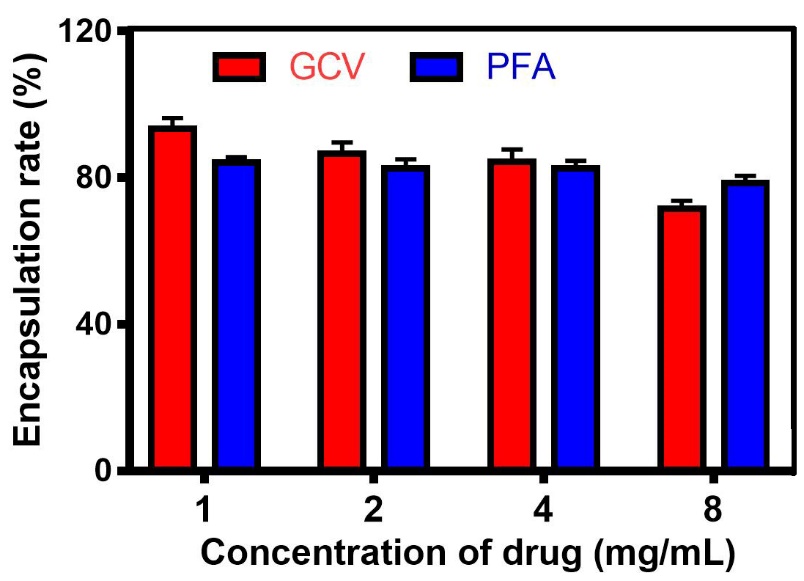


**Fig. S2.** The loading efficiency of PLGA/DOTAP nanoparticles. The dosage of PLGA was 20 mg (n=3).

**
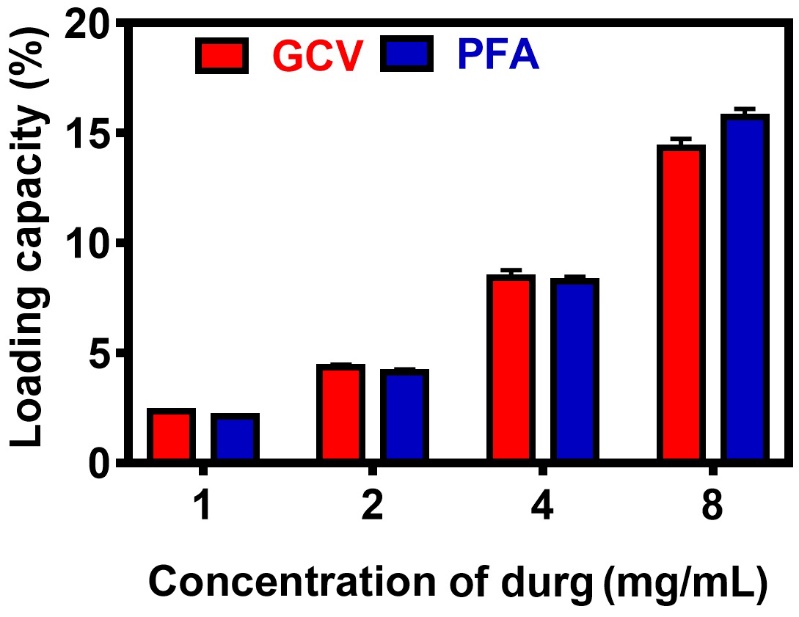
**

**Fig. S3.** The loading capacity of PLGA/DOTAP nanoparticles. The dosage of PLGA was 20 mg (n=3).

**
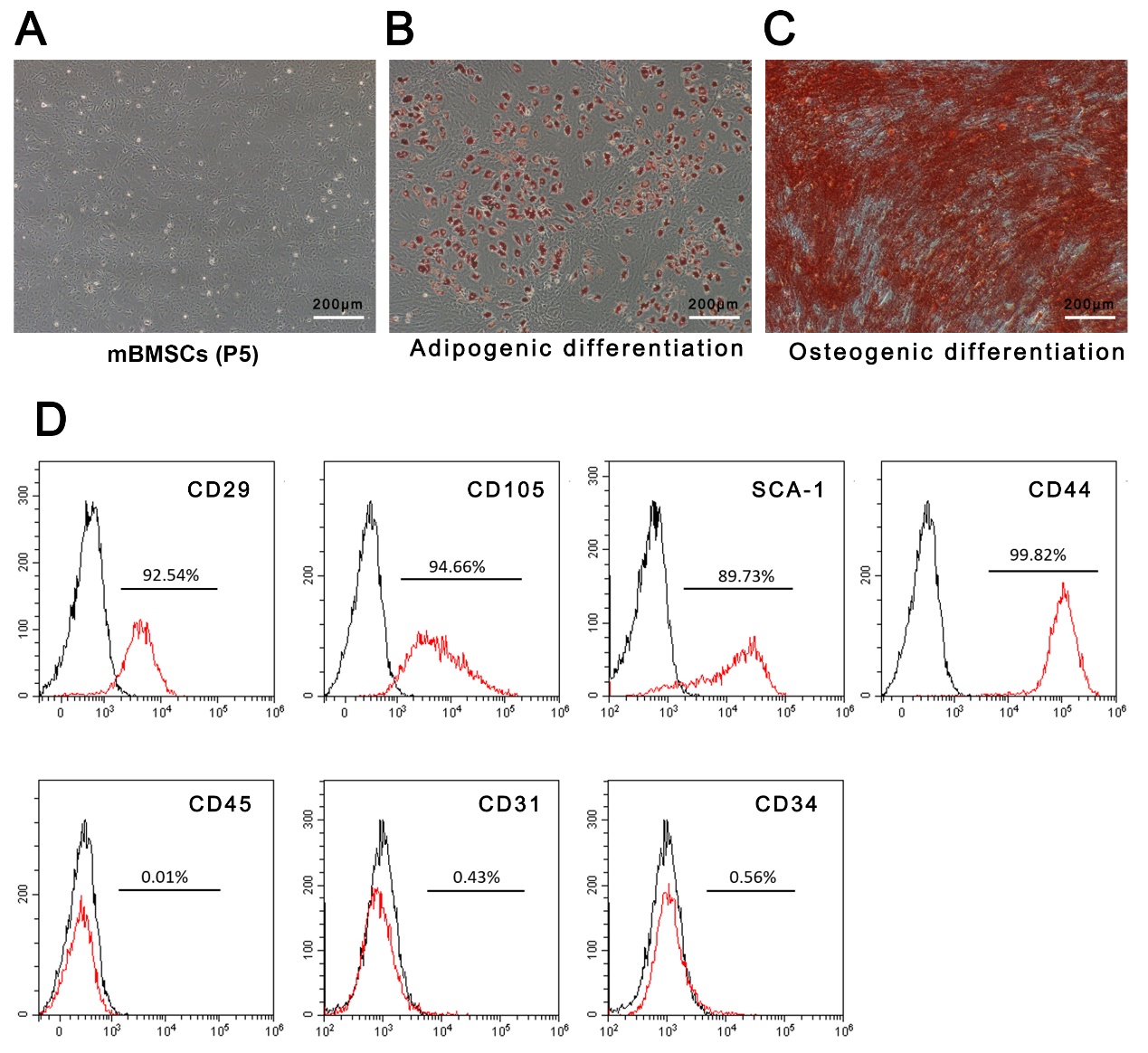
**

**Fig. S4**. The identifications of mBMSCs by FACS. (A) The morphology of mBMSCs at passages 5 (P5). (B) Adipocyte differentiation with Oil Red O staining. (C) Osteoblast differentiation with Alizarin red S staining. (D) FACS analysis of the surface markers of mBMSC. Among them, CD29, CD44, CD105, and SCA-1 were positively expressed, and CD45, CD31, and CD34 were negatively expressed.


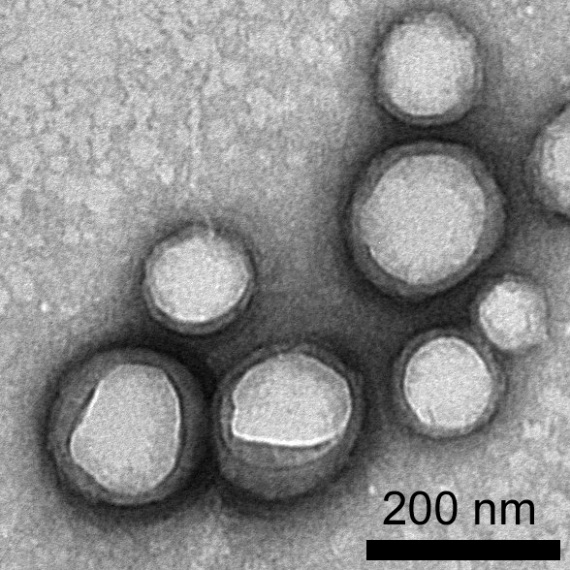


**Fig. S5**. TEM analysis of MPDGP.


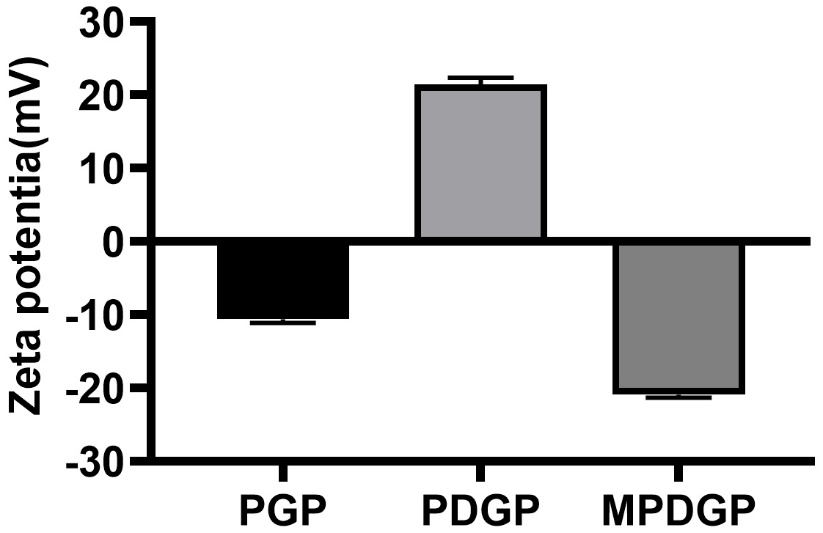


**Fig. S6** Zeta potential analysis of the nanoparticles.

**
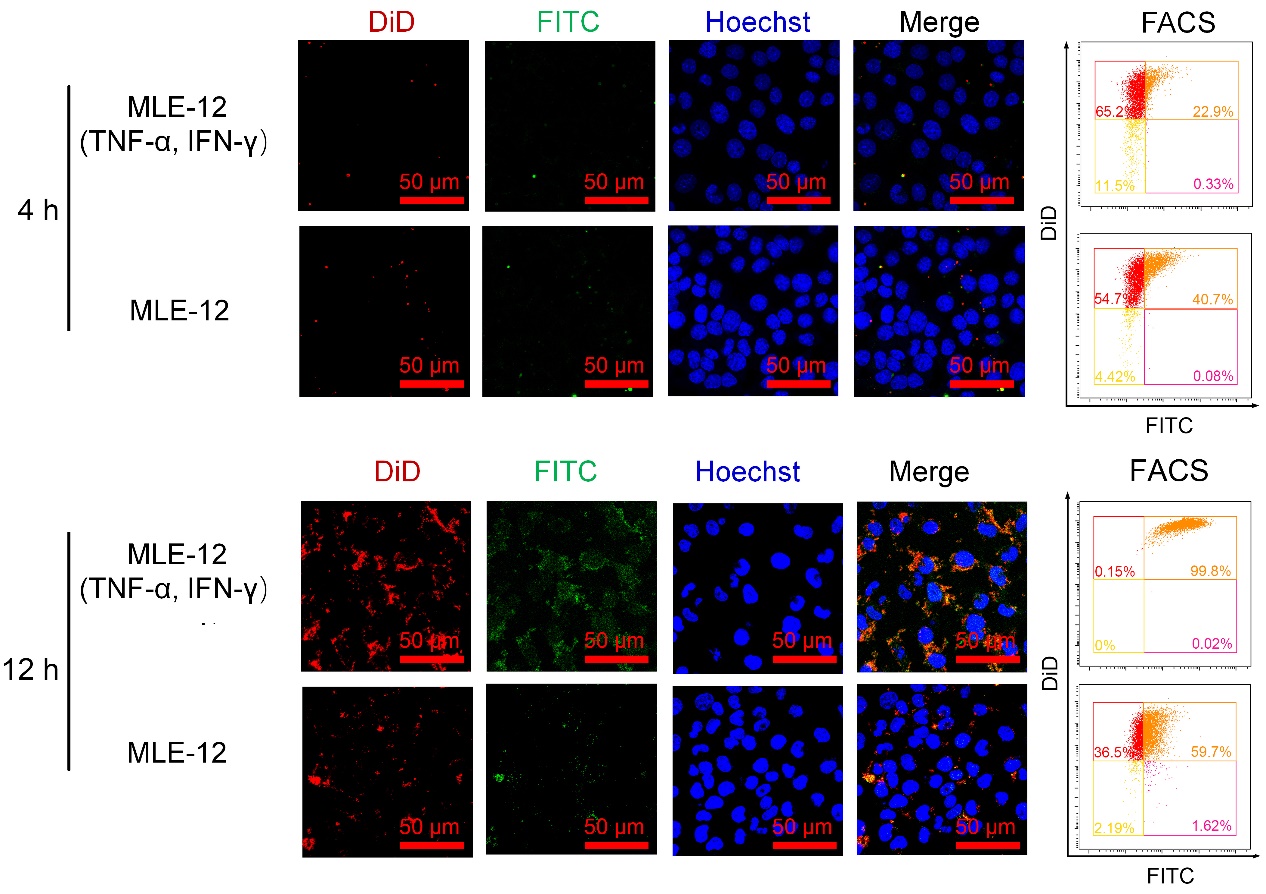
**

**Fig. S7.** Inflammatory tropism *in vitro*. MLE-12 cells were pretreated with or without TNF-α (10 ng/mL) and IFN-γ (10 ng/mL) for 24 h. The cells were treated with MDP/DiD/FITC for 4 h or 12 h. CLSM and FACS analysis were performed to evaluate the inflammatory tropism.


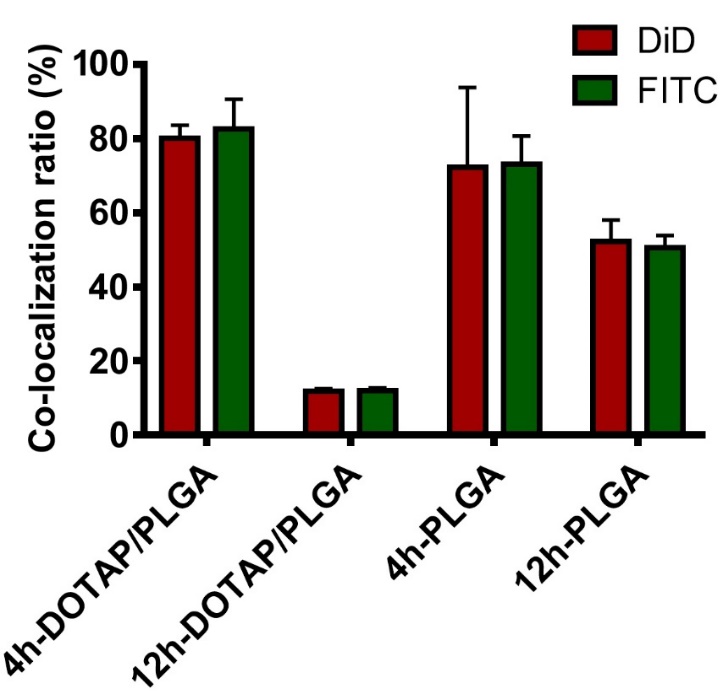


**Fig. S8.** The co-localization ratios analysis between components and lysosomes.
